# Gallstone Ileus: Management and Clinical Outcomes

**DOI:** 10.3390/medicina55090598

**Published:** 2019-09-17

**Authors:** Matas Jakubauskas, Raminta Luksaite, Audrius Sileikis, Kestutis Strupas, Tomas Poskus

**Affiliations:** 1 Clinic of Gastroenterology, Nephrourology and Surgery, Institute of Clinical Medicine, Faculty of Medicine, Vilnius University, 03101 Vilnius, Lithuania; 2 Center of Abdominal Surgery, Vilnius University Hospital “Santaros Klinikos”, 08410 Vilnius, Lithuania; 3 Department of Radiology, Nuclear Medicine and Physics of Medicine, Center for Radiology and Nuclear Medicine, Faculty of Medicine, Vilnius University, 03101 Vilnius, Lithuania; 4 Center of Radiology and Nuclear Medicine, Vilnius University Hospital “Santaros Klinikos”, 08410 Vilnius, Lithuania

**Keywords:** biliary ileus, gallstone, diagnosis, treatment, endoscopy, laparoscopy

## Abstract

*Background:* Gallstone or biliary ileus is a late complication of gallstone disease. It accounts for 1%–4% of all bowel obstructions and is more common in elderly patients. The preferred treatment option is to mechanically remove the impacted stones. It is done surgically using open or laparoscopic approach and rarely, when stones are impacted in the colon, endoscopically. In this paper we present five consecutive cases of gallstone ileus and describe possible diagnostic and minimally invasive treatment options. *Case presentation:* During a five-month period a total of five patients were treated for gallstone ileus. All patients were female and from 48 to 87 years of age. Symptoms were not specific and common for all small bowel obstructions. Upon admission the patients also had unspecific laboratory findings—neutrophilic leukocytosis and various C-reactive protein concentrations, ranging from 8 to 347 mg/L. According to the hospital protocol, all patients initially underwent an abdominal ultrasound, which was inconclusive, and therefore every patient additionally had a CT scan with intravenous contrast. After these two diagnostic modalities one patient still did not have the definitive gallstone ileus diagnosis, as the ectopic stone was not visible. Four patients in our case series were treated using minimally invasive methods: in one case the stone was removed endoscopically, and laparoscopically in the other three. Treatment outcomes were good in four cases as the patients fully recovered, however one patient suffered a massive cerebral infarction after the operation and passed away. *Conclusions:* Gallstone ileus is a rare and difficult-to-diagnose condition. Management of these patients in every case should be individualized, as there are many options, each with their own advantages and disadvantages. We show that minimally invasive treatment such as colonoscopy or laparoscopy is possible in these cases.

## 1. Introduction

Gallstone or biliary ileus is a late complication of gallstone disease. It accounts for 1%–4% of all bowel obstructions and is more common in elderly patients [1]. It occurs after the spontaneous formation of a biliary-enteric fistula, most commonly with the duodenum, less commonly with the colon, and very rarely with the stomach [2]. The preferred treatment option is to mechanically remove the impacted stones. This is done surgically using open or laparoscopic approaches and rarely, when stones are impacted in the colon, endoscopically [2,3,4,5]. In this paper we present five consecutive cases of gallstone ileus and describe possible diagnostic and minimally invasive treatment options.

## 2. Case Presentation

**Figure 1:**
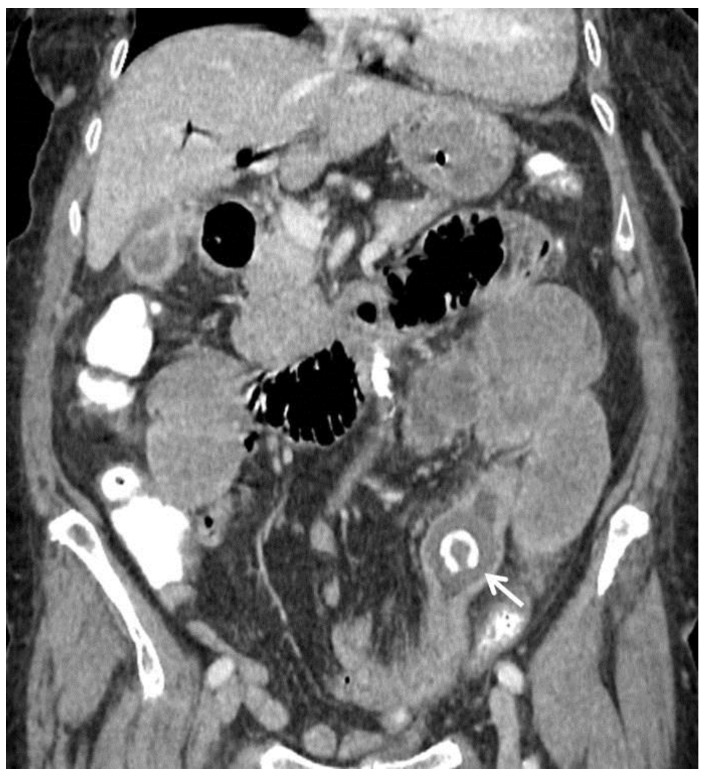
82-year-old female. Abdominal CT scan, coronal plane, portovenous phase. The transition point of the obstruction is visible, the gall stone is obstructing the small bowel lumen (arrow), distal loops are collapsed.

**Figure 2:**
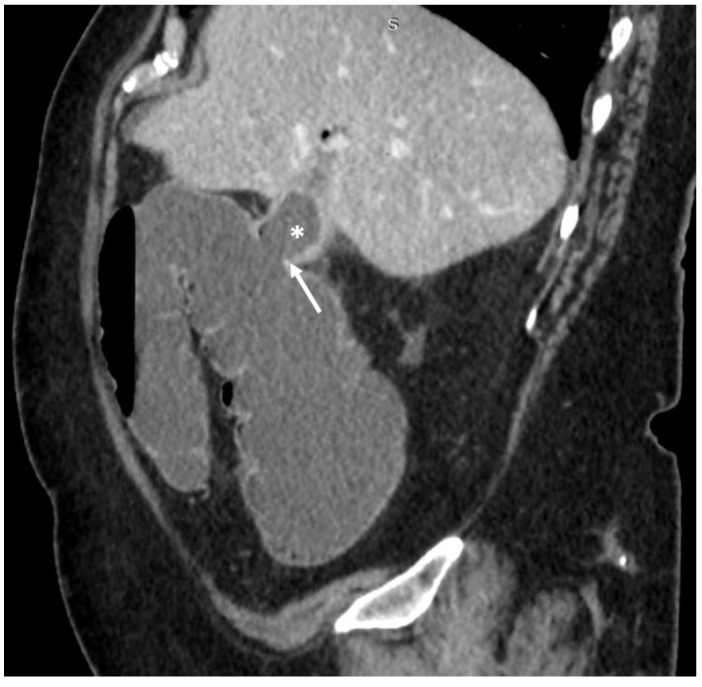
69-year-old female. Abdominal CT scan, sagittal plane, portovenous phase. Collapsed gallbladder (asterisk), there is a defect in the wall connecting the lumen of the gall bladder with the lumen of hepatic flexure of the colon–cholecystocolic fistula (arrow).

**Figure 3:**
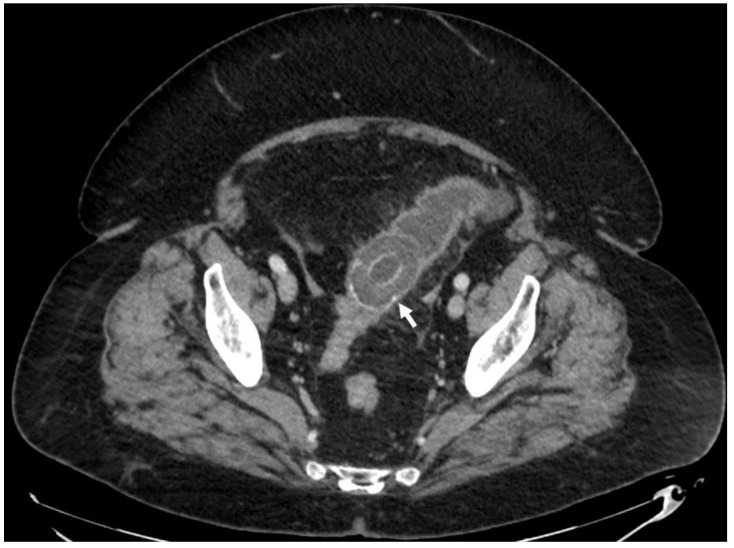
69-year-old female. Abdominal CT scan, axial plane, portovenous phase. The gallstone that migrated through cholecystocolic fistula is obstructing the lumen of sigma (arrow).

**Figure 4:**
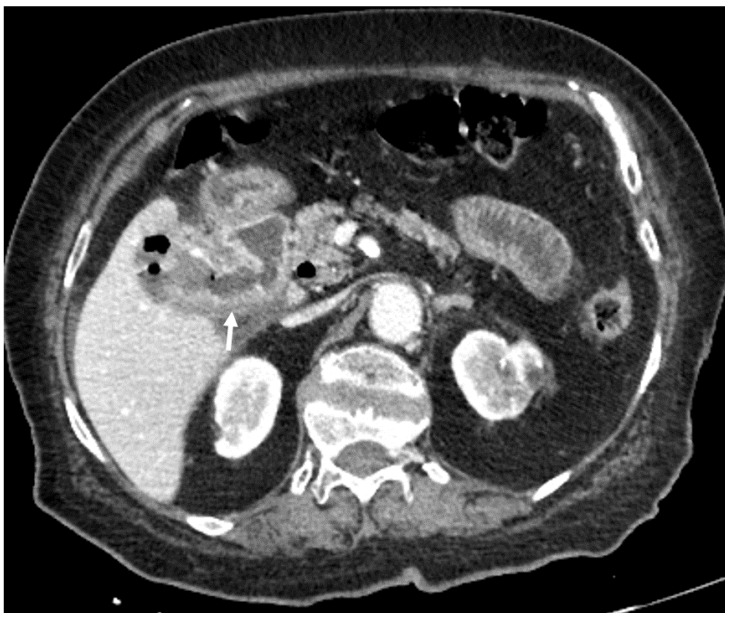
87-year-old female. Abdominal CT scan, axial plane, portovenous phase. Collapsed gallbladder, with thickened walls, pericholecystitis and air in the pericholecystic space. Also, a connecting path between the gallbladder and duodenum is visible (arrow)—cholecystoduodenal fistula.

**Figure 5:**
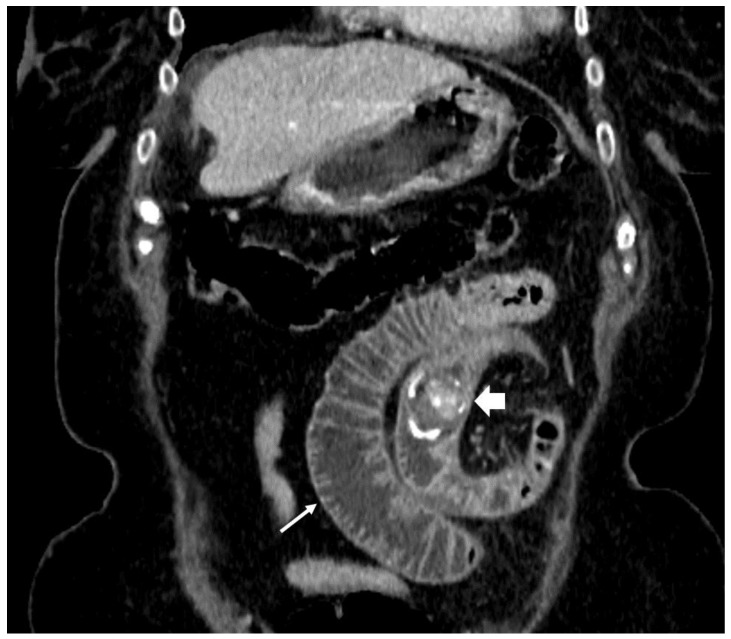
87-year-old female. Abdominal CT scan, coronal plane, portovenous phase. Dilated segment of proximal jejunal loops (thin arrow) caused by migrated gallstone (thick arrow) obstruction. Distal to the gallstone, jejunal loops are collapsed.

**Figure 6:**
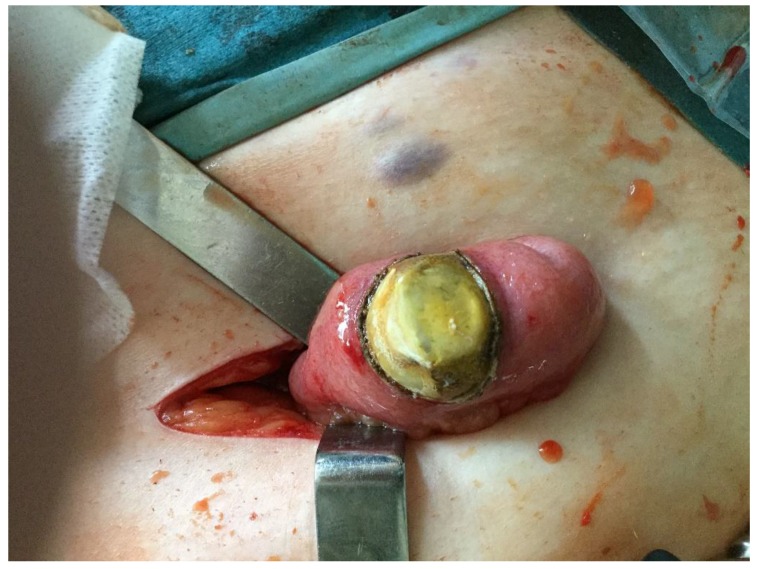
A 3 × 4 cm gallstone in the ileum, removed through a McBurney incision.

**Figure 7:**
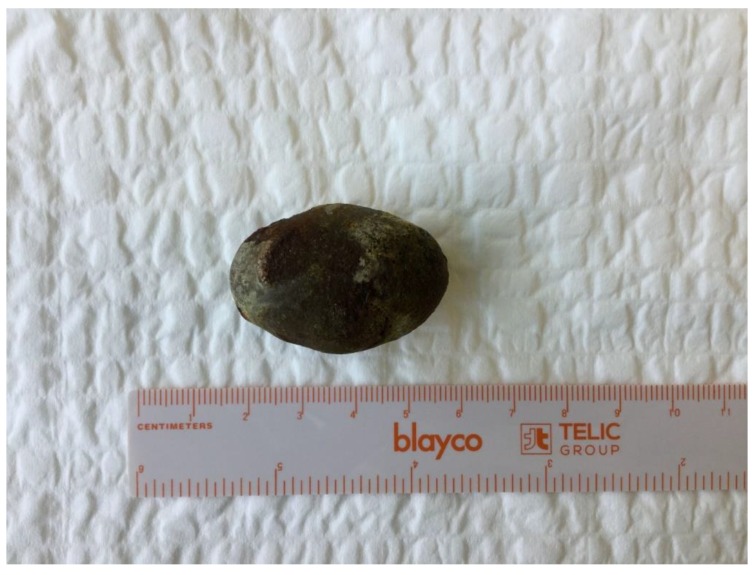
A 4 × 3 cm gallstone removed during laparoscopically assisted enterolithotomy.

**Figure 8:**
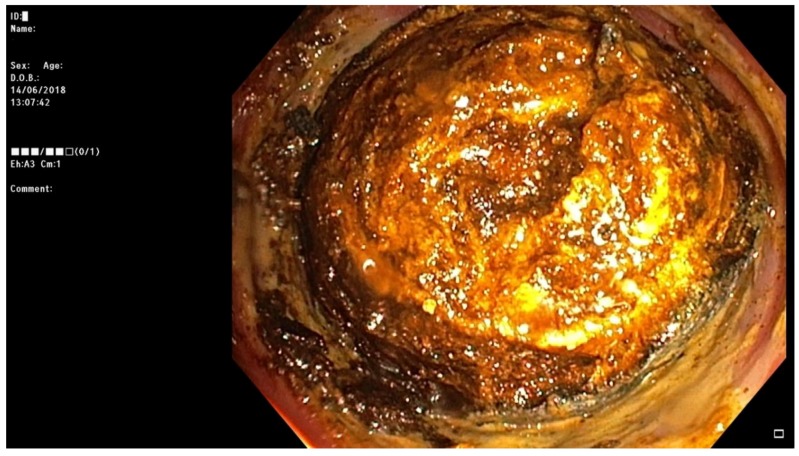
Impacted gallstone in the sigmoid colon successfully removed during colonoscopy.

During a five-month period between April 2018 and September 2018 a total of five patients were treated for gallstone ileus (Table 1). All patients were female with ages ranging from 48 to 87 years old. All procedures performed in studies involving human participants were in accordance with the ethical standards of the institutional and national research committee and with the 1964 Helsinki declaration and its later amendments or comparable ethical standards. All participants of this case series gave consent to publish clinical data and to use images representing their cases. Symptoms were not specific and are common for all small-bowel obstructions and include nausea, vomiting, abdominal distention, and pain. In one of the five reported cases the patient did not vomit; the remaining four patients all had bowel obstruction symptoms. Upon admission the patients also had unspecific laboratory findings—neutrophilic leukocytosis and various C-reactive protein concentrations, ranging from 8 to 347 mg/L. Additionally the three oldest patients had moderate or severe electrolyte imbalance. For the two oldest patients elevated lactate levels were found, suggesting a mesenteric artery thrombosis diagnosis, which was ruled out after a CT scan. According to the hospital protocol, all of our patients initially underwent an abdominal ultrasound, which was inconclusive. Therefore every patient additionally had a CT scan with intravenous contrast (Figure 1, Figure 2, Figure 3, Figure 4 and Figure 5). After these two diagnostic modalities one patient still did not have the definitive gallstone ileus diagnosis, as the ectopic stone was not visible. However, the presence of a biliary fistula and a history of stones in the gallbladder made the gallstone ileus diagnosis most probable. In our cases conservative treatment was the first line therapy for all patients except for one, and even though the impacted stones were not very big, this approach was not effective and patients ultimately required surgical or endoscopic stone removal procedures. Four patients in our case series were treated using minimally invasive methods: in one case the stone was removed endoscopically, and laparoscopically in the other three (Figure 6, Figure 7 and Figure 8). In one case, due to the serious condition of the patient and surgeons’ preference, the operation was started with a laparotomy. During the hospitalization the three oldest patients were admitted to the intensive care unit, as one developed renal failure and the other two had unstable hemodynamics. Treatment outcomes were good in four cases, as the patients fully recovered, but one patient suffered a massive cerebral infarction after the operation and passed away a day later.

## 3. Discussion

Biliary, or gallstone, ileus more frequently occurs in elderly (>70 years) female patients with a history of cholelithiasis and several comorbidities [6,7,8]. In our case, all patients were elderly women, and two of them had a history of complicated gallstone disease. The stones causing obstruction originate from the gallbladder through an enteric fistula, however biliary ileus in patients with no fistula or gallbladder have been documented [9,10]. Most of the time the stone gets impacted in the terminal ileum, then in the colon and very rarely in the stomach, causing a Bouveret gastric outlet syndrome [2,11].

Imaging is key for the diagnosis of gallstone ileus. Rigler’s triad on plain abdominal radiograms of air–fluid levels, pneumobilia, and an ectopic stone has been described [12]. Gallstones are often radiolucent. The cause of obstruction often remains undetermined on abdominal radiograms, and the full triad is found in only 14%–53% of cases [6,13,14,15]. Other imaging options such as CT scans and ultrasound perform better [13,16,17,18]. The use of ultrasound enables the detection not only of the impacted stone, but also the site of the fistula and the presence of cholelithiasis [17]. Ultrasound is highly operator-dependent and the technical difficulties when performing an ultrasound on a patient with bowel obstruction (discomfort, bowel full of gas/fluid) make it difficult to use as the main modality in the acute abdomen setting [16]. Therefore, the CT scan is presently considered to be the gold standard for diagnosing gallstone ileus [14,16,19]. CT scan has the highest sensitivity for the Rigler’s triad signs. It also allows pinpointing of the place of obstruction, cholecystoenteric fistula, and the more accurate investigation of the ectopic stone size [14,16,19]. These advantages grant earlier diagnosis and help with more accurate patient management [14,16,19].

If the general condition is stable, it is possible to start the treatment conservatively (especially if the gallstones are less than 2.5 cm), albeit the spontaneous resolution rates are low [20,21,22,23]. There is a broad spectrum of approaches for achieving obstruction relief described in the literature, ranging from exotic and relatively uncommon methods such as shockwave lithotripsy [24,25] and lithotripsy using Nd:YAG laser [26] to more clinically available, but still niche methods (i.e., endoscopic removal) [3], and to the most popular but also most invasive surgical approach [4,7,27]. There is a great deal of controversy regarding the surgical procedure of choice for gallstone ileus [1,7,11,28,29]. The debate goes on as to which of three possible procedures (one-stage, two-stage, or just enterolithotomy) to use. The one-stage procedure addresses all problems at once by including the enterolithotomy, cholecystectomy, and fistula closure. The two-stage procedure also addresses all pathologies at once but within a longer time period, as the cholecystectomy with fistula closure is done 4–6 weeks after the enterolithotomy. Currently the most widely used procedure is enterolithotomy alone [1,8,11]. Reisner et al. reported higher mortality rates in the one-stage procedure group (16.9%) compared to enterolithotomy alone (11.7%) [11]. The main drawback of enterolithotomy is the unaddressed biliary fistula, which later on may cause recurrent cholangitis, sepsis, or even gallstone ileus recurrence [11,29]. However, there are reports of spontaneous fistula closure, thus eliminating the need for the fistula closure in the first place [30]. The recurrence of gallstone ileus after enterolithotomy is rare, around 5%–9%, and only about 10% of these patients require reoperation [11,31,32,33]. Although the mortality has shown to be quite similar between these two approaches, the morbidity may still be higher in the one-stage method group, thus leaving the one-stage procedure for highly selected patients [7,34,35].

Colonoscopy can only be rarely used, as the stone needs to be in the colon, and is unreachable otherwise. The laparoscopic approach is more versatile; it can be used to remove stones not only from the small bowel, as in our series, but also from the sigmoid colon [4]. The biggest drawback is the difficulty in detecting the obstructing gallstone. Once identified, it should be marked and its proximal migration should be avoided. Al Skaini et al. did not detect the stone during laparoscopy, and it was found 3 days later at laparotomy [36]. 

## 4. Conclusions

Gallstone ileus is a rare and a difficult-to-diagnose condition. Management of these patients in every case should be individualized, as there are many options, each with their own advantages and disadvantages. We show that minimally invasive treatment such as colonoscopy or laparoscopy is possible in these cases.

## Figures and Tables

**Table 1:** General demographics, treatment details, and outcomes for patients with gallstone ileus.

Gender, Age	Time from Hospital Admission to Stone Removal (hours)	ASA Class	Procedure	Procedure Time (min)	Impacted Gallstone Location	Impacted Gallstone Size	Length of Treatment in ICU (days)	Overall Hospital Stay (days)	Outcomes
F, 82	25	III	Laparoscopically assisted enterolithotomy	95	Ileum	3 × 3 cm	3	14	Good: recovery
F, 48	40	III	Laparoscopically assisted enterolithotomy	70	Ileum	3 × 4 cm	-	10	Good: recovery
F, 85	47	IV	Laparoscopically assisted enterolithotomy	60	Ileum	4 × 3 cm	12	13	Good: recovery
F, 69	12	III	Colonoscopy	115	Sigmoid colon	5 × 4 cm	-	10	Good: recovery
F, 87	2	IV	Laparotomy with enterolithotomy	35	Jejunum	4 × 3 cm	5	7	Poor: death

ASA: American Society of Anesthesiologists; ICU: intensive care unit.

## Abbreviations

CT: computed tomography.
